# Age-Adjusted Endothelial Activation and Stress Index for Coronavirus Disease 2019 at Admission Is a Reliable Predictor for 28-Day Mortality in Hospitalized Patients With Coronavirus Disease 2019

**DOI:** 10.3389/fmed.2021.736028

**Published:** 2021-09-08

**Authors:** Felipe Pérez-García, Rebeca Bailén, Juan Torres-Macho, Amanda Fernández-Rodríguez, Maria Ángeles Jiménez-Sousa, Eva Jiménez, Mario Pérez-Butragueño, Juan Cuadros-González, Julen Cadiñanos, Irene García-García, María Jiménez-González, Pablo Ryan, Salvador Resino

**Affiliations:** ^1^Unidad de Infección Viral e Inmunidad, Centro Nacional de Microbiología, Instituto de Salud Carlos III, Madrid, Spain; ^2^Servicio de Microbiología Clínica, Hospital Universitario Príncipe de Asturias, Madrid, Spain; ^3^Servicio de Hematología y Hemoterapia, Hospital General Universitario Gregorio Marañón, Madrid, Spain; ^4^Instituto de Investigación Sanitaria Gregorio Marañón, Madrid, Spain; ^5^Servicio de Medicina Interna, Hospital Universitario Infanta Leonor, Madrid, Spain; ^6^Servicio de Medicina Preventiva, Hospital Universitario Infanta Leonor, Madrid, Spain; ^7^Servicio de Pediatria, Hospital Universitario Infanta Leonor, Madrid, Spain; ^8^Departamento de Biomedicina y Biotecnología, Facultad de Medicina, Universidad de Alcalá de Henares, Madrid, Spain; ^9^Servicio de Medicina Interna, Hospital General de Villalba, Collado Villalba, Spain; ^10^Servicio de Farmacología Clínica, Hospital Universitario La Paz-IdiPAZ, Madrid, Spain; ^11^Departamento de Medicina, Facultad de Medicina, Universidad Complutense de Madrid, Madrid, Spain

**Keywords:** COVID-19, mortality, clinical prediction rule, blood coagulation disorders, endothelium

## Abstract

**Background:** Endothelial Activation and Stress Index (EASIX) predict death in patients undergoing allogeneic hematopoietic stem cell transplantation who develop endothelial complications. Because coronavirus disease 2019 (COVID-19) patients also have coagulopathy and endotheliitis, we aimed to assess whether EASIX predicts death within 28 days in hospitalized COVID-19 patients.

**Methods:** We performed a retrospective study on COVID-19 patients from two different cohorts [derivation (*n* = 1,200 patients) and validation (*n* = 1,830 patients)]. The endpoint was death within 28 days. The main factors were EASIX [(lactate dehydrogenase ^*^ creatinine)/thrombocytes] and aEASIX-COVID (EASIX ^*^ age), which were log_2_-transformed for analysis.

**Results:** Log_2_-EASIX and log_2_-aEASIX-COVID were independently associated with an increased risk of death in both cohorts (*p* < 0.001). Log_2_-aEASIX-COVID showed a good predictive performance for 28-day mortality both in the derivation cohort (area under the receiver-operating characteristic = 0.827) and in the validation cohort (area under the receiver-operating characteristic = 0.820), with better predictive performance than log_2_-EASIX (*p* < 0.001). For log_2_ aEASIX-COVID, patients with low/moderate risk (<6) had a 28-day mortality probability of 5.3% [95% confidence interval (95% CI) = 4–6.5%], high (6–7) of 17.2% (95% CI = 14.7–19.6%), and very high (>7) of 47.6% (95% CI = 44.2–50.9%). The cutoff of log_2_ aEASIX-COVID = 6 showed a positive predictive value of 31.7% and negative predictive value of 94.7%, and log_2_ aEASIX-COVID = 7 showed a positive predictive value of 47.6% and negative predictive value of 89.8%.

**Conclusion:** Both EASIX and aEASIX-COVID were associated with death within 28 days in hospitalized COVID-19 patients. However, aEASIX-COVID had significantly better predictive performance than EASIX, particularly for discarding death. Thus, aEASIX-COVID could be a reliable predictor of death that could help to manage COVID-19 patients.

## Introduction

Around 80% of severe acute respiratory syndrome coronavirus 2 (SARS-CoV-2)-infected patients develop mild-to-moderate illness, 15% severe illness, and 5% critical illness, including acute respiratory distress syndrome, septic shock, and multiorgan failure (1). Severe coronavirus disease 2019 (COVID-19) is related to high mortality, mostly in older people with comorbidities such as diabetes and cardiovascular diseases (2). Besides, the excessive hospital demand generated by the COVID-19 pandemic during the first wave caused a high request for intensive care beds in Madrid, Spain (3), affecting the quality of medical care and impacting mortality due to COVID-19 (4).

A deregulated pro-inflammatory response (cytokine storm) usually appears in patients with severe COVID-19, which leads to coagulopathy and endothelial damage with frequent episodes of thromboembolism (1, 5). Widespread endotheliitis with diffuse microcirculatory injury in the lung and other organs (brain, heart, kidneys, gut, and liver) is a central feature of severe COVID-19 (1). This disturbed coagulation is strongly associated with acute respiratory distress syndrome, multiorgan failure, and mortality, which is higher than in patients with COVID-19-unrelated pneumonia (1).

During the COVID-19 pandemic, many biomarkers to predict mortality have been reported (6, 7), including lactate dehydrogenase, creatinine, and thrombocyte count. These three markers are part of the Endothelial Activation and Stress Index (EASIX), a powerful score that was initially developed to predict survival in patients with acute graft vs. host disease (GVHD) after allogeneic hematopoietic stem cell transplantation (allo-HSCT) (8). Endothelial activation is the common trigger of several complications occurring after allo-HSCT, including transplant-associated microangiopathy, sinusoidal obstruction syndrome, and GVHD (9). In the last years, EASIX has also been validated as a predictor for the development of other allo-HSCT complications, including non-relapse mortality (10, 11), fluid overload (12), and sinusoidal obstruction syndrome (13). This score has also been validated in other hematological malignancies outside of the HSCT setting (14, 15).

Because coagulopathy and endothelial dysfunction are critical in the evolution of patients with COVID-19, we aimed to assess whether the EASIX score can predict 28-day mortality in hospitalized COVID-19 patients.

## Methods

### Patients

We performed a retrospective study on consecutively hospitalized patients between March 1 and May 31, 2020 (during the first wave of the COVID-19 pandemic) with a laboratory-confirmed with a laboratory-confirmed SARS-CoV-2 infection by real-time polymerase chain reaction. Our study population consisted of two cohorts from two hospitals in Madrid, Spain, which were previously described:

(i) *Derivation cohort* from Infanta Leonor University Hospital (ILUH) (16, 17). Initially, 1,968 patients were included. However, we discarded 391 patients due to missing values for the EASIX variables and 377 patients due to transfer to another institution within 28 days after hospital admission, resulting in a final study population of 1,200 patients. The Ethics Committee of ILUH (Code ILUH R 027-20) approved the study.

(ii) *Validation cohort* from La Paz University Hospital (LPUH) (18). Initially, 2,226 patients were included. We discarded 396 patients due to missing values for the EASIX variables, resulting in a final study population of 1,830 patients. The Ethics Committee of LPUH (Code PI-4072) approved the study.

The study was conducted according to the Declaration of Helsinki. Written informed consent waiver was obtained from the Ethics Committees due to the retrospective nature of the study. In addition, the database was anonymized for statistical analysis. The research followed the Transparent Reporting of a Multivariable Prediction Model for Individual Prognosis or Diagnosis (TRIPOD) statement (19).

### Clinical Data

Demographic and clinical data were extracted from medical records and managed using Research Electronic Data Capture (REDCap). We included age, sex, smoking habit, comorbidities [chronic heart disease, hypertension, chronic pulmonary disease, asthma, chronic kidney disease, liver disease (cirrhosis), neoplasm, hematological malignancy, obesity, diabetes, and dyslipidemia], laboratory findings, and signs at hospital admission [oxygen saturation, hematocrit, blood counts (lymphocytes, neutrophils, thrombocytes), aspartate aminotransferase and alanine aminotransferase, lactate dehydrogenase, glucose, creatinine, sodium, potassium, and C-reactive protein].

EASIX was calculated according to the previously reported formula [lactate dehydrogenase (IU/L) ^*^ creatinine (mg/dl)/thrombocyte count (10^9^ cells/L)] (8). Additionally, we calculated the aEASIX-COVID (age-adjusted EASIX for COVID-19), which incorporates age at COVID-19 diagnosis to the previous formula [EASIX ^*^ age (years)]. Age was added to EASIX because it is a significant predictor of mortality in COVID-19 patients (20) and is also an easy variable to obtain at the time of the patient's diagnosis. Both indexes were log_2_ transformed.

### Outcome Variables

The primary endpoint was 28-day all-cause mortality. The baseline was the date at hospital admission. At the follow-up censoring date (May 31, 2020), the clinical status of the patients was discharged alive, currently hospitalized alive, or dead. When a patient was readmitted during the study period, a single hospital admission episode was considered for the purposes of the analysis.

### Statistical Analysis

Quantitative variables were expressed as the median and interquartile range, and categorical variables were shown as absolute count (percentage). Comparisons between groups were performed using the Mann–Whitney U test for continuous variables and the chi-squared or two-tailed Fisher's exact test for categorical variables.

We assessed the risk of death using the survival analysis (Kaplan–Meier and Cox regression analyses). The Kaplan–Meier product-limit method was used to estimate survival probabilities at 28 days, and the log-rank test was used to calculate the differences between groups and trends. Cox proportional-hazards models were used to study the association between risk factors (age, sex, smoking habit, comorbidities, laboratory findings, and signs at hospital admission) and mortality during the first 28 days. Continuous variables (including EASIX and aEASIX-COVID) were log_2_-transformed (base-2 logarithms). First, we performed univariate Cox regression analyses. Then, we performed multivariate Cox regression analyses with variables that had a *p*-value ≤ 0.05, missing values ≤10%, and low collinearity between them (*r* < 0.5), which were further selected by a stepwise forward selection method (pin < 0.05 and pout < 0.10).

Internal validation of the predictive model was made using 20-fold cross-validation. The predictive performance of death within 28 days of hospital admission for EASIX and aEASIX-COVID was evaluated by examining calibration (Hosmer–Lemeshow test) and discrimination [area under the receiver-operating characteristic (AUROC)] measures. We calculated the prediction error for EASIX and aEASIX-COVID in both cohorts using the Brier score. Differences between AUROC models were assessed using the Delong test. Sensitivity, specificity, positive predictive value (PPV), and negative predictive value (NPV) were calculated for the different deciles of the distribution.

Statistical analysis was performed using Stata/IC 15.1 (StataCorp, Texas, USA) and GraphPad Prism 7.04 (GraphPad Software, Inc., California, USA).

## Results

### Patient Characteristics

Table 1 shows baseline characteristics of COVID-19 patients, stratified by survival/death within 28 days of hospital admission at ILUH (derivation cohort) and LPUH (validation cohort). In both cohorts, patients who died were significantly older, more frequently male, and presented more comorbidities such as chronic heart disease, hypertension, chronic kidney disease, solid neoplasm, hematological malignancy, diabetes, and dyslipidemia. Besides, patients who died showed significantly lower values of hematocrit, lymphocytes, thrombocytes, and alanine aminotransferase, whereas they had higher values of neutrophils, aspartate aminotransferase, lactate dehydrogenase, glucose, creatinine, potassium, and C-reactive protein. Mortality rate within 28 days was significantly lower in ILUH (derivation cohort, 17.7%) than in LPUH (validation cohort, 22.5%) (*p* = 0.001).

**Table 1:** Baseline characteristics of hospitalized COVID-19 patients, stratified by survival at 28 days after admission.

**Characteristic**	**Survivors**	**Non-survivors**	***p*-value**
**A) ILUH (derivation cohort)**			
No. patients	988 (82.3%)	212 (17.7%)	–
Age, median (IQR)	64 (52–77)	82 (72–87)	**<0.001**
Sex (male)	562 (65.9%)	152 (71.7%)	**<0.001**
**Comorbidities**			
Chronic heart disease	182 (18.7%)	90 (42.5%)	**<0.001**
Hypertension	486 (49.9%)	152 71.7%)	**<0.001**
Chronic pulmonary disease	106 (10.9%)	50 (23.9%)	**<0.001**
Asthma	85 (8.7%)	11 (5.2%)	0.052
Chronic kidney disease	50 (5.1%)	30 (14.3%)	**<0.001**
Liver cirrhosis	14 (1.4%)	7 (3.4%)	0.062
Neoplasm	32 (3.4%)	29 (12.7%)	**<0.001**
Hematological malignancy	18 (1.9%)	13 (5.6%)	**0.004**
Obesity	154 (18.6%)	26 (15.0%)	0.327
Diabetes	249 (21.4%)	73 (31.6%)	**0.001**
Dyslipidemia	220 (22.7%)	67 (31.8%)	**0.005**
Smoker	50 (7.4%)	9 (7.7%)	0.850
**Laboratory findings and signs**			
Oxygen saturation in room air (%)	95 (92–97)	90 (82–93)	**<0.001**
Hematocrit (%)	41.5 (38.4–44.2)	39.4 (35.0–43.7)	**<0.001**
Lymphocyte count (cells/μl)	1,000 (800–1,400)	800 (500–1,100)	**<0.001**
Neutrophil count (cells/μl)	4,800 (3,500–6,900)	5,900 (3,800–8,450)	**<0.001**
Thrombocyte count (x 10^9^ cells/L)	212 (165–275)	187 (137–264)	**<0.001**
Aspartate Aminotransferase (IU/L)	37 (27–53)	47 (31–71)	**<0.001**
Alanine Aminotransferase (IU/L)	36 (26–56)	31 (22–48)	**<0.001**
Lactate dehydrogenase (IU/L)	255 (207–327)	353 (265–480)	**<0.001**
Glucose (mg/dL)	109 (98–132)	135 (110–167)	**<0.001**
Creatinine (mg/dL)	0.99 (0.80–1.20)	1.31 (1.03–1.94)	**<0.001**
Sodium (mEq/L)	139 (136–141)	138 (136–142)	0.715
Potassium (mEq/L)	4.2 (3.9–4.6)	4.4 (4.0–4.8)	**0.004**
C-reactive Protein (mg/L)	60.9 (22.7–122.0)	119.8 (56.2–208.7)	**<0.001**
**B) LPUH (validation cohort)**			
No. patients	1,418 (77.5%)	412 (22.5%)	–
Age, median (IQR)	65 (53–77)	81 (74–87)	**<0.001**
Sex (male)	745 (52.6%)	264 (64.1%)	**<0.001**
**Comorbidities**			
Chronic heart disease	281 (19.9%)	161 (36.4%)	**<0.001**
Hypertension	660 (46.7%)	278 (67.5%)	**<0.001**
Chronic pulmonary disease	108 (7.7%)	41 (10.1%)	0.124
Asthma	72 (5.1%)	12 (2.9%)	0.081
Chronic kidney disease	95 (6.7%)	80 (19.5%)	**<0.001**
Liver cirrhosis	15 (1.1%)	7 (1.7%)	0.305
Neoplasm	151 (10.7%)	86 (21.0%)	**<0.001**
Hematological malignancy	88 (6.2%)	53 (12.9%)	**<0.001**
Obesity	238 (17.2%)	67 (16.8%)	0.880
Diabetes	284 (20.1%)	125 (30.5%)	**<0.001**
Dyslipidemia	530 (37.6%)	212 (51.6%)	**<0.001**
Smoker	100 (7.3%)	36 (9.8%)	0.111
**Laboratory findings and signs**			
Oxygen saturation in room air (%)	91.9 (75.9–95.3)	92.4 (82.2–96.4)	0.258
Hematocrit (%)	42.3 (38.9–45.1)	40.5 (36.3–44.4)	**<0.001**
Lymphocyte count (cells/μl)	1,130 (790–1,690)	710 (470–1,060)	**<0.001**
Neutrophil count (cells/μl)	3,845 (2,830–5,550)	6,135 (3,870–9,085)	**<0.001**
Thrombocyte count (x 10^9^ cells/L)	249 (191–322)	217 (161–290)	**<0.001**
Aspartate Aminotransferase (IU/L)	31 (21–50)	41 (26–60)	**<0.001**
Alanine Aminotransferase (IU/L)	32 (21–55)	27 (18–45)	**<0.001**
Lactate dehydrogenase (IU/L)	286 (226–361)	385 (293–514)	**<0.001**
Glucose (mg/dl)	99 (88–116)	115 (97–149)	**<0.001**
Creatinine (mg/dl)	0.77 (0.63–0.93)	1.02 (0.77–1.49)	**<0.001**
Sodium (mEq/L)	139 (137–142)	140 (136–143)	0.060
Potassium (mEq/L)	4.0 (3.7–4.3)	4.1 (3.7–4.5)	**0.008**
C-reactive Protein (mg/L)	32.7 (6.5–96.5)	126.8 (56.4–209.6)	**<0.001**

*Statistics: Values are expressed as median and interquartile range for continuous variables and absolute count (percentage) for categorical variables. p-values were calculated by chi-squared or two-tailed Fisher's exact test for categorical variables and Mann–Whitney test for continuous variables. Significant differences are shown in bold. Abbreviations: p-value, level of significance; IU, international units; ul, microliter; ILUH, Infanta Leonor University Hospital; LPUH, La Paz University Hospital*.

### Risk of Death Within 28 Days

Log_2_ EASIX was associated with a higher risk for death within 28 days in the derivation cohort [adjusted hazard ratio (aHR) = 1.55; *p* < 0.001] and the validation cohort (aHR = 1.41; *p* < 0.001) (Supplementary Table 1). Furthermore, log_2_ aEASIX-COVID showed slightly higher mortality risk values for 28-day death compared with log_2_ EASIX (Supplementary Table 2), both in the derivation (aHR = 1.61; *p* < 0.001) and in the validation cohort (aHR = 1.51; *p* < 0.001).

### Predictive Performance of Death Within 28 Days

**Figure 1:**
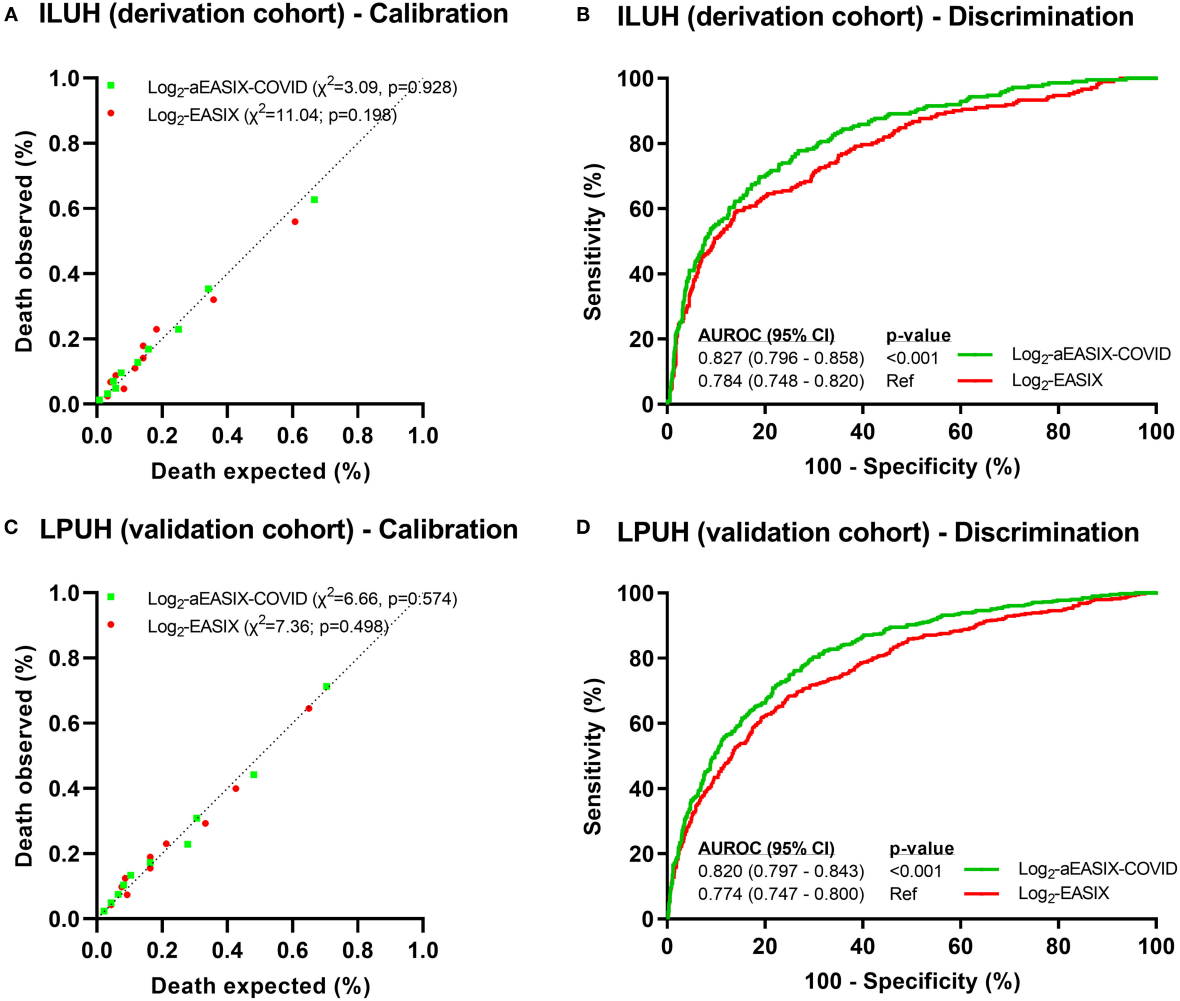
Predictive performance of death within 28 days in COVID-19 patients. Calibration plots **(A,C)** were performed from Hosmer–Lemeshow test. Discrimination analysis was performed by AUROC curves **(B,D)**, and *p*-values were calculated using Delong test. Abbreviations: X^2^, Chi-squared; AUROC, area under the receiver-operating characteristic curve; 95% CI: 95% confidence interval; EASIX, endothelial activation and stress index; aEASIX-COVID: age-adjusted EASIX at COVID-19 diagnosis.

Log_2_ EASIX presented suitable values of calibration (chi-squared = 11.04; *p* = 0.198; Figure 1A), discrimination (AUROC = 0.784; Figure 1B), and an acceptable prediction error (Brier score = 0.119) at the derivation cohort. At the validation cohort, log_2_ EASIX showed similar predictive performance values to the derivation cohort for calibration (chi-squared = 7.36; *p* = 0.498; Figure 1C), discrimination (AUROC = 0.774; Figure 1D), and an admissible prediction error (Brier score = 0.141). Log_2_ EASIX PPV increased with deciles but did not exceed 61% in the derivation cohort and 70% in the validation cohort, and NPV decreased with the increase of the deciles but was not <80% in both cohorts (Supplementary Table 3).

Log_2_ aEASIX-COVID showed better values of predictive performance than log_2_ EASIX for calibration and discrimination in the derivation cohort [chi-squared = 3.09 (*p* = 0.928; Figure 1A) and AUROC = 0.827 (*p* < 0.001; Figure 1B), respectively] and in the validation cohort [chi-squared = 6.66 (*p* = 0.574; Figure 1C) and AUROC = 0.820 (*p* < 0.001; Figure 1D), respectively]. Moreover, Brier scores of log_2_ aEASIX-COVID were slightly lower than those obtained for log_2_ EASIX (0.111 for derivation and 0.131 for validation cohorts). Internal validation showed an AUC of 0.832 (95% CI = 0.786–0.849) in the derivation cohort and 0.818 (95% CI = 0.795–0.842) in the validation cohort. Log_2_ aEASIX-COVID PPV raised with the increase in deciles but did not exceed 67% in the derivation cohort and 72% in the validation cohort. Besides, NPV decreased with increasing deciles but was not below 80% in both cohorts (Supplementary Table 4).

### Probability of Death Within 28 Days

**Figure 2:**
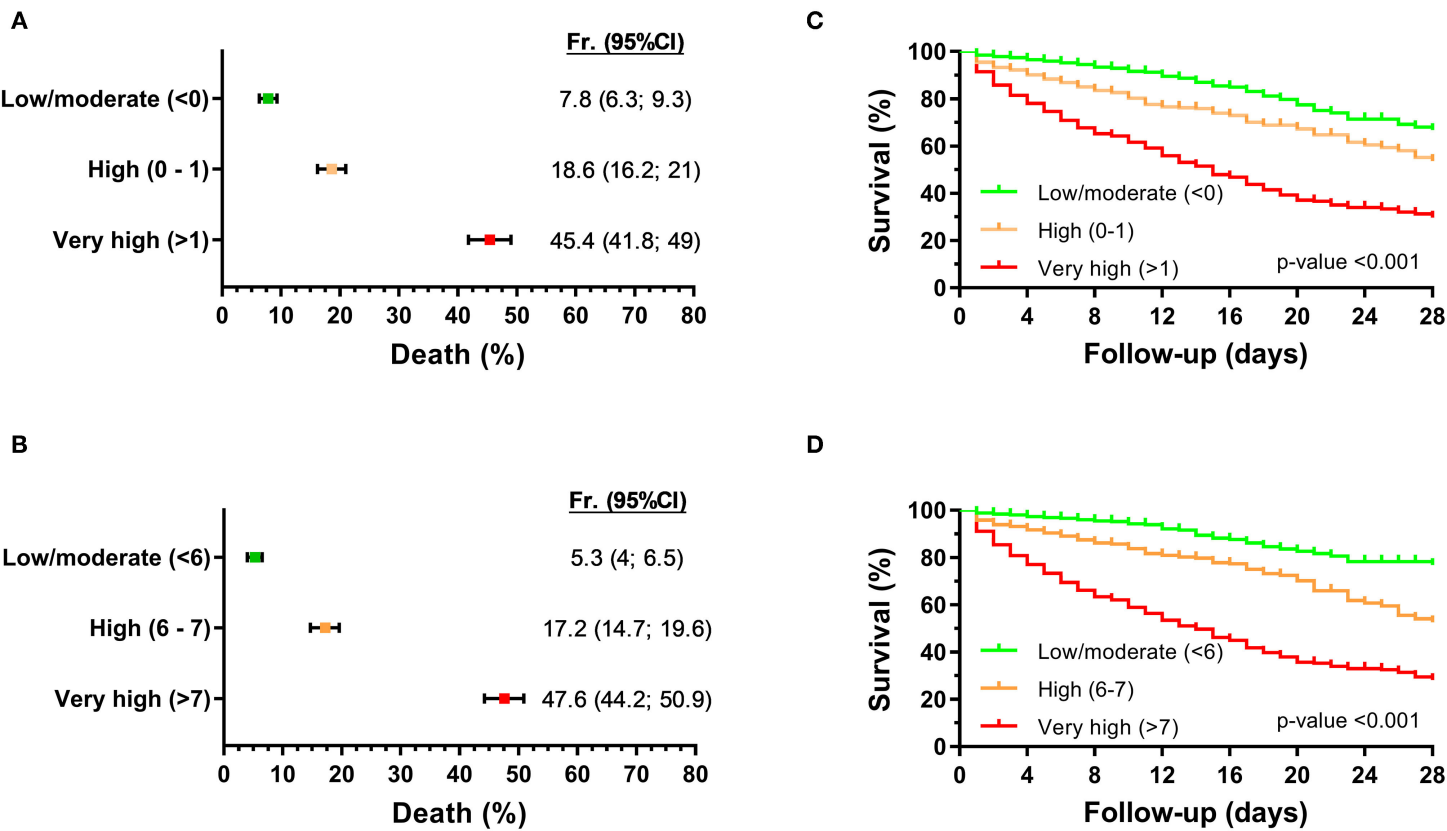
Prediction of 28-day mortality in hospitalized COVID-19 patients according to log_2−_aEASIX and log_2−_aEASIX-COVID stratified into six risk categories. **(A,B)** probability of death within 28 days of hospitalization according to log_2−_aEASIX and log_2−_aEASIX-COVID, respectively. Values are expressed as frequency and 95% confidence interval (95% CI). **(C,D)** Survival curves (Kaplan-Meier curve) by log_2−_aEASIX and log_2−_aEASIX-COVID risk categories, respectively. *P*-value was calculated by log-rank trend tests. Abbreviations: Fr, frequency; 95% CI, 95% confidence interval; EASIX, endothelial activation and stress index; aEASIX-COVID, age-adjusted EASIX at COVID-19 diagnosis.

We considered the risk of 28-day mortality, joining the two cohorts, in three strata (low/moderate, high, and very high). For log_2_ EASIX, 28-day mortality probability values were 7.8% for patients with low/moderate risk (<0), 18.6% for high risk (0–1), and 45.4% for very high risk (>1) (Figure 2A). The cutoff of log_2_ EASIX = 0 showed a PPV of 29.8% and NPV of 92.2%, and log_2_ EASIX = 1 showed a PPV of 45.3% and NPV of 87.4% (Table 2). For log_2_ aEASIX-COVID, 28-day mortality probability values were 5.3% for patients with low/moderate risk (<6), 17.2% for high risk (6, 7), and 47.6% for very high risk (>7) (Figure 2B). The cutoff of log_2_ aEASIX-COVID = 6 showed a PPV of 31.7% and NPV of 94.7%, and log_2_ aEASIX-COVID = 7 showed a PPV of 47.6% and NPV of 89.8% (Table 2). The Kaplan–Meier curve for the 28-day mortality also showed a different evolution of patients according to the different risk strata according to log_2_ EASIX (Figure 2C) and log_2_ aEASIX-COVID (Figure 2D).

**Table 2:** Sensitivity, specificity, PPV, and NPV for predicting 28-day mortality in hospitalized COVID-19 patients according to log_2−_EASIX and log_2_-aEASIX-COVID deciles.

**Cutoff**	**Risk of 28-day death**	**Sensitivity (95% CI)**	**Specificity (95% CI)**	**PPV (95% CI)**	**NPV (95% CI)**
**log2 EASIX**					
0	Low/moderate	84.1 (81.0–86.9)	48.5 (46.5–50.6)	29.8 (27.7–32.0)	92.2 (90.6–93.6)
1	Very high	53.8 (49.8–57.8)	83.2 (81.6–84.6)	45.3 (41.7–49.0)	87.4 (86.0–88.8)
**log2 aEASIX-COVID**					
6	Low/moderate	89.3 (86.6–91.6)	50.2 (48.1–52.2)	31.7 (29.5–34.0)	94.7 (93.4–95.9)
7	Very high	64.3 (60.4–68.0)	81.7 (80.1–83.2)	47.6 (44.2–51.1)	89.8 (88.5–91.0)

*Abbreviations: EASIX, Endothelial Activation and Stress Index; aEASIX-COVID, age-adjusted EASIX for COVID-19; PPV, positive predictive value; NPV, negative predictive value; 95% CI, 95% confidence interval*.

## Discussion

We evaluated EASIX for predicting mortality within 28 days in hospitalized COVID-19 patients from two large datasets in Spain. The main findings of our study were as follows: (i) the increase in EASIX values, especially in aEASIX-COVID, was linked to higher 28-day mortality. (ii) EASIX and aEASIX-COVID had a good predictive performance, but only aEASIX-COVID had AUROC >0.8 in the derivation and validation cohorts. (iii) EASIX and aEASIX-COVID were more reliable in predicting patient survival than death because the NPV values were much higher than the PPV values. (iv) EASIX and aEASIX-COVID allowed the stratification of COVID-19 patients into three risk categories of 28-day mortality.

Many predictive scores for mortality in COVID-19 patients have been developed (20, 21). However, most of these predictive scores do not exceed the AUROC of 0.8, including comorbidities related to poor COVID-19 prognosis or variables that are not always available in clinical practice. Besides, these scores require laborious calculations as long as they are based on complex multivariate models. Therefore, we hypothesized that EASIX, a simple score developed for endotheliopathy associated with allo-HSCT, could also predict mortality in COVID-19 patients because endotheliopathy is crucial for its pathophysiology (1).

EASIX was initially developed by Luft et al. as a predictor of survival in patients with acute GVHD after allo-HSCT (8) and later validated to predict mortality related to different post-HSCT complications (10–15). For the development of EASIX, the authors chose three laboratory parameters that were part of the classical diagnostic criteria of thrombotic microangiopathy (creatinine, lactate dehydrogenase, and thrombocyte counts) due to both their simplicity and their association with endothelial dysfunction and microangiopathy (8). Widespread endotheliitis and coagulopathy are also keys in the pathophysiology of COVID-19 (1). Recently, Luft et al. (22) have reported in two cohorts of 100 and 126 patients that EASIX predicts COVID19 outcome and may discriminate patients who need intensive surveillance. Besides, high EASIX values correlated with increased serum values of endothelial (angiopoietin-2, CXCL8, soluble thrombomodulin, and suppressor of tumorigenicity-2) and inflammatory (CXCL9, IL18, and IL18BPa) biomarkers (22). In our study, EASIX showed reasonable accuracy in predicting death within 28 days in hospitalized COVID-19 patients despite its simplicity and the fact that it was developed in a different setting. Both the simplicity and applicability make this score especially useful in healthcare overload and low-resource settings.

Moreover, because age has largely been described as one of the most important predictors of mortality in patients with COVID-19 (20), we postulated that an age-adjusted EASIX (aEASIX-COVID) might increase its predictive performance for 28-day mortality. In our study, the predictive performance of the aEASIX-COVID was significantly superior to the EASIX (initial model) in our two cohorts (derivation and validation) (23). However, EASIX and aEASIX-COVID were more reliable in predicting patient survival than death because NPV values were much higher than PPV values. Furthermore, the predictive performance of aEASIX-COVID for 28-day mortality was similar to Sociedad Española de Enfermedades Infecciosas y Microbiología Clínica (SEIMC) (24) and PANDEMYC scores (17), which were both constructed from patients included in our study. However, SEIMC and PANDEMYC scores are more complex to calculate because they are constructed with a higher number of variables (seven to nine variables) than aEASIX-COVID (four variables), and their developments were based on more complex calculations.

Our study presents some limitations. First, this retrospective study only included patients belonging to the first pandemic wave, which was associated with higher mortality rates worldwide. Another limitation could be that aEASIX-COVID relied exclusively on hospitalized patients. Consequently, its applicability in primary care settings, where routine laboratory tests are not usually used, is unknown. Finally, a limitation common to all reported COVID-19 prognostic models is that our study was carried out in Spain, limiting our findings' extrapolation to other countries and healthcare settings. In this regard, the level of hospital saturation generated in the first wave of the COVID-19 epidemic could affect our results (new admissions, number of transfers to other hospitals daily, patient/physician ratio, available intensive care unit beds, among others). Consequently, additional studies are needed to validate the diagnostic performance of aEASIX-COVID in different epidemiological contexts. Further complementary studies could include the evaluation of EASIX and aEASIX-COVID for the prediction of cardiovascular and thromboembolic complications (such as pulmonary thromboembolism) in the context of COVID-19.

This study also has several strengths. First, our research has a large sample size and a large number of events, both in the derivation and validation cohorts. Besides, our research adheres to the TRIPOD recommendations. Finally, the aEASIX-COVID score is easy to calculate with normally accessible variables, which would allow rapid decision-making in COVID-19 patients.

## Conclusion

Both EASIX and aEASIX-COVID were associated with death within 28 days in hospitalized COVID-19 patients. However, aEASIX-COVID had significantly better predictive performance than EASIX, particularly for discarding death. Thus, our findings suggest that aEASIX-COVID could be a reliable predictor of death that could help to manage COVID-19 patients.

## Data Availability Statement

The datasets used and/or analyzed during the current study may be available from the corresponding author upon reasonable request.

## Ethics Statement

The studies involving human participants were reviewed and approved by Infanta Leonor University Hospital Ethics Committee (Code: ILUH R 027-20) and the Ethics Committee of La Paz University Hospital (Code: PI-4072). Written informed consent for participation was not required for this study in accordance with the national legislation and the institutional requirements.

## Author Contributions

SR and PR: funding body. FP-G and SR: study concept, design, statistical analysis, and interpretation of data. PR, JT-M, EJ, MP-B, JC, J-CG, IG-G, and MJ-G: patients' selection and clinical data acquisition. FP-G, RB, and SR: writing of the manuscript. PR, MÁJ-S, and AF-R: critical revision of the manuscript for relevant intellectual content. SR: supervision and visualization. All authors contributed to the article and approved the submitted version.

## Funding

This study was supported by grants from Instituto de Salud Carlos III [grant number COV20/1144 [MPY224/20) to AF-R/MÁJ-S]. MÁJ-S and AF-R are supported by Instituto de Salud Carlos III (grant numbers CP17CIII/00007 and CP14CIII/00010, respectively).

## Conflict of Interest

PR reports grants and personal fees from GILEAD and MSD and personal fees from AbbVie and ViiV Healthcare, outside the submitted work. SR reports reports grants from GILEAD and MSD, outside the submitted work. The remaining authors declare that the research was conducted in the absence of any commercial or financial relationships that could be construed as a potential conflict of interest.

## Publisher's Note

All claims expressed in this article are solely those of the authors and do not necessarily represent those of their affiliated organizations, or those of the publisher, the editors and the reviewers. Any product that may be evaluated in this article, or claim that may be made by its manufacturer, is not guaranteed or endorsed by the publisher.

## Collaborators

A) *Derivation cohort from Infanta Leonor University Hospital (ILUH)*: Ruth Solana; Samuel Manzano; Raquel Ruiz; Raquel Barba; Pilar Tejedor; Muria Muñoz-Rivas; Mercedes Drake; Belen Mateo; Marta Vara; Marta Alvarado; Jorge Valencia; Mario Fontan; Laura Zazo; Natalia Blanca; Isabel Torres; Ines Fernandez; Ana Tebar; Alba Bergaz; Ana Prieto; Andrea Lazaro; Adriana Campoverde; Beatriz Mestre; Beatriz Fernandez; Gerardo Redondo; Guillermo Cuevas; Mariano Matarranz; Berta Montero; Elsa Izquierdo; Helena Notario; Beatriz Sanchez; Ana Such; Elena Alba Alvaro; Virginia Pardo; Mateo Balado; Maria Gonzalez; Isamel Escobar; Carlos Bibiano; Paz Arranz; Francisco Ceballos; Eva Moya.

B) *Validation cohort from La Paz University Hospital (LPUH)*: José Ramón Arribas; Alberto M. Borobia; Antonio Carcas-Sansuán; Jesús Frías; Elena Ramírez; Alejandro Martín-Quirós; Manuel Quintana-Díaz; Jesús Mingorance; Francisco Arnalich; Francisco Moreno; Juan Carlos Figueiras; Nicolás García-Arenzana; María Dolores Montero Vega; María Pilar Romero Gómez; Carlos Toro-Rueda; Silvia García-Bujalance; Guillermo Ruiz-Carrascoso; Emilio Cendejas-Bueno; Iker Falces-Romero; Fernando Lázaro-Perona; Mario Ruiz-Bastián; Almudena Gutiérrez-Arroyo; Patricia Girón De Velasco-Sada; Elie Dahdouh; Bartolomé Gómez-Arroyo; Consuelo García-Sánchez; Virginia Guedez-López; Iván Bloise-Sánchez; Marina Alguacil-Guillén; Maria Gracia Liras-Hernández; Miguel Angel Sánchez-Castellano; Paloma García-Clemente; Patricia González-Donapetry; Sol San José-Villar; Manuela de Pablos Gómez; Rosa Gómez-Gil; Maria Teresa Corcuera-Pindado; Alicia Rico-Nieto; Alicia Herrero; Daniel Prieto Arribas; Paloma Oliver-Saez; Roberto Mora Corcovado; Pilar Fernández-Calle; Mª José Alcaide Martín; Jorge Díaz-Garzón Marco; Belén Fernández-Puntero; Rocío Nuñez Cabetas; Gema Crespo Sánchez; Olaia Rodriguez Fraga; Helena Mendez del Sol; Marta Duque Alcorta; Rubén Gomez Rioja; María Sanz de Pedro; Lydia Pascual García; Marta Segovia Amaro; Jose Manuel Iturzaeta Sánchez; Mercedes Rodriguez Gutiérrez; Amparo Perez Garcia Morillon; Miguel Angel Martinez Gallego; Blanca Fabre Estremera; Estefaní Martinez; Isabel Moreno Parra; Neila Rodriguez Roca; Daniel Ortiz Sánchez; Manuela Simon Velasco; Ileana Gabriela Tomoiu; Cristina Pizarro Sanchez; Blanca Montero San Martín; Ana Laila Qasem Moreno; Marta Gómez López; Ismael Casares Guerrero; Antonio Buño Soto; Milagros Martí de Gracia; Luz Parra Gordo; Aurea Diez Tascón; Silvia Ossaba Vélez; Inmaculada Pinilla; Emilio Cuesta; María Fernández-Velilla; Maria Isabel Torres; Gonzalo Garzón.; Verónica Pérez-Blanco; Almudena Quintás-Viqueira; Isabel San Juan; José Miguel Cantero-Escribano; César Pérez-Romero; Mercedes Castro-Martínez; Lucia Hernández-Rivas; Teresa Pedraz; Eva Fernández-Bretón; Claudia García-Vaz; Ana Robustillo-Rodela; Rosario María Torres Santos-Olmo; Angélica Rivera Núñez; Ignacio Fernández Osaba; Marina Noguerol Gutiérrez; Ana María Martínez Virto; Manuel González Viñolis; Regina Cabrera Gamero; Rosa Mayayo Alvira; Raquel Marín Baselga; Victoria Lo-Iacono García; Macarena Lerín Baratas; Paloma Romero Gallego-Acho; Begoña Reche Martínez; Renzo Tejada Sorados; Mikel Rico Briñas; Ricardo Deza Palacios; Sara Fabra Cadenas; Isabel Arroyo Rico; Lubna Dani Ben-Abdellah; Laura Labajo Montero; Rubén Soriano Arroyo; Lorena López Corcuera; Elena Calvin García; Susana Martínez Álvarez; Laura López-Tappero Irazábal; Martín Pilares Barco; Olga González Peña; Guillermina Bejarano Redondo; Alberto Iglesias Sigüenza; Yale Tung Chen; Charbel Maroun Eid; Ruth Bravo Lizcano; Miguel Silvestre Niño; Frank Perdomo García; Berta Alonso González; Berta Antón Huguet; Isabel Arenas Berenguer; Clara Cabré-Verdiell Surribas; Francisco Marqués González; Elena Muñoz Del Val; María Ángeles Molina; Nataly Cancelliere Fernández; Sivia Pastor Yvorra; Laura Frade Pardo; Paloma López Arévalo; Isabel García; Carmen Fernández Capitán; Juan José González Garcia; Juan Herrero; María Angustias Quesada Simón; Angel Robles Marhuenda; Clara Soto Abanedes; Ana María Noblejas Mozo; Juan Carlos Ramos; Maria Jesús Jaras Hernandez; Elena Martinez Robles; Alberto Moreno Fernandez; Aquilino Sanchez Purificación; Juan Carlos Martin Gutiérrez; Pedro Luis Martinez Hernández; Teresa Sancho Bueso; Alicia Lorenzo Hernández; Belén Gutierrez Sancerni; Giorgina Salgueiro; Luz Martin Carbonero; Jose mAría Mostaza; María Angeles Martinez-López; Victor Hontañon; Araceli Menéndez; Jorge Alvarez Troncoso; Arancha Castellano; Cristina Marcelo Calvo; Ivo Vives Beltrán; Luis Ramos Ruperto; German Daroca Bengoa; María Arcos Rueda; Julia Vasquez Manau; Pelayo Fernández Cidón; Carmen Rosario Herrero Gil; Esmeralda Palmier Peláez; Yeray Untoria Tabares; Carlos Lahoz; Eva Estirado; Clara Hernández; Francisca Garcia-Iglesias; Enrique Monteoliva; Mónica Martínez; Marta Varas; Teresa González Alegre; Maria Eulalia Valencia; Victoria Moreno; Maria Luisa Montes.; Sergio Alcolea Batres; Juan José Cabanillas Martín; Carlos Carpio Segura; Raquel Casitas Mateo; Jaime Fernández-Bujarrabal Villoslada; Isabel Fernández Navarro; Juan Fernández Lahera; Cristina García Quero; María Hidalgo Sánchez; Raúl Galera Martínez; Francisco García Río; Luis Gómez Carrera; María Antonia Gómez Mendieta; Alberto Mangas Moro; Elisabet Martínez Cerón; María Martínez Redondo; Yolanda Martínez Abad; Antonio Martínez-Verdasco; Cristina Plaza Moreno; Sarai Quirós Fernández; Delia Romera Cano; David Romero Ribate; Begoña Sánchez Rebate; Ana Santiago Recuerda; Carlos Villasante Fernández-Montes; Ester Zamarrón De Lucas; Victoria Arnalich Montiel; Pablo Mariscal Aguilar; Adalgisa Falcone; Daniel Laorden Escudero; María Concepción Prados Sánchez; Rodolfo Álvarez-Sala Walther; Andony García; Cristina Arévalo; Carola Gutiérrez; Santiago Yus; Maria José Asensio; Manolo Sánchez; Jose Manuel Añón; Jesús Manzanares; Abelardo García De Lorenzo; Eva Perales; Belén Civantos; Lucía Cachafeiro; Alexander Agrifoglio; Belén Estébanez; Eva Flores; Mónica Hernández; Pablo Millán; Montserrat Rodríguez; Kapil Nanwani; Beatriz Arizcun; Elena Pérez-Costa; Diego Rodríguez-Álvarez; María Sánchez-Martín; Úrsula Quesada; Carmen Román-Hernández; Paloma Dorao; Elena Álvarez-Rojas; Juan José Menéndez; Cristina Verdú; Ana Gómez-Zamora; Cristina Schüffelmann; Belén Calderón-Llopis; María Laplaza-González; Miguel Río-García; Irene Amores-Hernández; Miguel Rodríguez-Rubio; Pedro de la Oliva; Jose Ruiz; Sandra Rosillo; Oscar González; Angel Iniesta; Ines Ponz.; José María Muñoz Ramón; María Carmen Hernández Gancedo; Rafael Uña Orejón; Pascual Sanabria Carretero; Isidro Moreno Gómez-Limón; Alverio Seiz-Martinez; Emilia Guasch-Arévalo; Cristina Martín-Carrasco; Elena Alvar; Lucía Serrá; Fabricio Iannuccelli; Julieta Latorre; Sandra Casares; Isabel Valbuena; Luis Díaz Díez Picazo; Cristina Rodríguez Roca; Omar Cervera; Esteban García de las Heras; Pilar Durán; Carmen Castro; Carlos Manrique de Lara; Javier Veganzones; Araceli López-Tofiño; Estefanía Fernandez-Cerezo; Sergio Zurita; Mercedes López-Martinez; Teresa Prim; Julía Alvárez Del Vayo; Gabriela Alcaraz; Luis Castro; Julio Yagüe; Sofía Díaz-Carrasco; Patricio González-Pizarro; Ana Montero; Francisco Javier Sagra; Alejandro Suárez.; Leyre Díez Porres; María Varela Cerdeira; Alberto Alonso Babarro; Francisco Abellán Martínez; Jorge Ignacio Alonso Eiras; Alejandra Álvarez Brandt; Martina Archinà; Silvia Arribas Terradillos; Trinidad Baselga Puente; Pilar Barco Núñez; Natalia Guadalupe Barrera López; Lorena Barrera López; Andres Bartrina Tarrio; Gemma Bassani; Paula Betancort De la Torre; Irene Blanco Bartolomé; Celia Blasco Andres; Lucia Brieba Plata; Fernando Cadenas Gota; Paloma Carrera Vázquez; Carlota Cascajares Sanz; Arianna Catino; Raquel Cavallé Pulla; Daniel Ceniza Pena; Ylenia María Conde Alonso; Laura Currás Sánchez; Marcelo Daltro Lage; Ana Esteban Romero; María Luisa Fernández Vidal; Inés Ferrer Ortiz; Lydia de la Fuente Regaño; Pablo Galindo Ballesteros; Sara Garcia-Bellido Ruiz; Carlos García-Mochales Fortún; Teresa Gómez Ballesteros; Cecilia Gómez Domínguez; Nelsa González Aguado; Sofía González García; Jorge Guisández Martín; Paula Alejandra Hernández Liebo; Raquel Hernando Nieto; Irene María Llorente Cortijo; Antonio Marín García; Pilar López Pirez; Lucía Mejuto Illade; Marco Palma; Adrian Peña Hidalgo; Lucía Platero Dueñas; David Pujol Pocull; Miguel Ramírez Verdyguer; Marta Redondo Gutierrez; Francisco Reinoso Lozano; Ana Rodríguez Revillas; Alejandro Rodríguez Saenz de Urturi; Lucía Romero Imaz; Susana Sánchez Rico; Mónica Sánchez Santiuste; Patricia Serrano de la Fuente; Henar Serrano Martín; Thamires Silva Freire; Eva Soria Alcaide; Andrés Enrique Suárez Plaza; Beatriz Tejero Soriano; Andrea Torrecillas Mainez; Javier Torres Cortés; María de Las Mercedes Valentín-Pastrana Aguilar; Angélica Villanueva Freije; Marta Virgós Varela; Marta Yagüe Barrado; Natalia Yustas Benitez.; Mª Concepción Núñez; Jaime Montserrat; Javier Queiruga; Amelia Rodriguez Mariblanca; Lucía Martínez de Soto; Mikel Urroz; Enrique Seco; Mónica Zubimendi; Stephan Stuart; Lucía Díaz; Irene García.; María Teresa García Morales; Alberto Martín-Vega; Abel Caro; Gonzalo Martínez-Alés.

## References

[1] Osuchowski MF Winkler MS Skirecki T Cajander S Shankar-Hari M Lachmann G. The COVID-19 puzzle: deciphering pathophysiology and phenotypes of a new disease entity. Lancet Respir Med. (2021) 9:622–42. 10.1016/S2213-2600(21)00218-6 33965003 PMC8102044

[2] Zhou M Zhang X Qu J. Coronavirus disease 2019 (COVID-19): a clinical update. Front Med. (2020) 14:126–35. 10.1007/s11684-020-0767-8 32240462 PMC7115348

[3] Sanchez-Ubeda EF Sanchez-Martin P Torrego-Ellacuria M Rey-Mejias AD Morales-Contreras MF Puerta JL. Flexibility and bed margins of the community of madrid's hospitals during the first wave of the SARS-CoV-2 Pandemic. Int J Environ Res Public Health. (2021) 18:3510. 10.3390/ijerph18073510 33800638 PMC8036372

[4] Sen-Crowe B Sutherland M McKenney M Elkbuli A. A closer look into global hospital beds capacity and resource shortages during the COVID-19 pandemic. J Surg Res. (2021) 260:56–63. 10.1016/j.jss.2020.11.062 33321393 PMC7685049

[5] Bermejo-Martin JF Almansa R Torres A Gonzalez-Rivera M Kelvin DJ. COVID-19 as a cardiovascular disease: the potential role of chronic endothelial dysfunction. Cardiovasc Res. (2020) 116:e132–3. 10.1093/cvr/cvaa140 32420587 PMC7314234

[6] Izcovich A Ragusa MA Tortosa F Lavena Marzio MA Agnoletti C Bengolea A. Prognostic factors for severity and mortality in patients infected with COVID-19: a systematic review. PLoS ONE. (2020) 15:e0241955. 10.1371/journal.pone.0241955 33201896 PMC7671522

[7] Khodeir MM Shabana HA Alkhamiss AS Rasheed Z Alsoghair M Alsagaby SA. Early prediction keys for COVID-19 cases progression: a meta-analysis. J Infect Public Health. (2021) 14:561–9. 10.1016/j.jiph.2021.03.001 33848885 PMC7934660

[8] Luft T Benner A Jodele S Dandoy CE Storb R Gooley T. EASIX in patients with acute graft-versus-host disease: a retrospective cohort analysis. Lancet Haematol. (2017) 4:e414–23. 10.1016/S2352-3026(17)30108-4 28733186

[9] Pagliuca S Michonneau D Sicre de Fontbrune F Sutra Del Galy A Xhaard A Robin M. Allogeneic reactivity-mediated endothelial cell complications after HSCT: a plea for consensual definitions. Blood Adv. (2019) 3:2424–35. 10.1182/bloodadvances.2019000143 31409584 PMC6693000

[10] Luft T Benner A Terzer T Jodele S Dandoy CE Storb R. EASIX and mortality after allogeneic stem cell transplantation. Bone Marrow Transplant. (2020) 55:553–61. 10.1038/s41409-019-0703-1 31558788 PMC8082940

[11] Shouval R Fein JA Shouval A Danylesko I Shem-Tov N Zlotnik M. External validation and comparison of multiple prognostic scores in allogeneic hematopoietic stem cell transplantation. Blood Adv. (2019) 3:1881–90. 10.1182/bloodadvances.2019032268 31221661 PMC6595255

[12] Varma A Rondon G Srour SA Chen J Ledesma C Champlin RE. Endothelial activation and stress index (EASIX) at admission predicts fluid overload in recipients of allogeneic stem cell transplantation. Biol Blood Marrow Transplant. (2020) 26:1013–20. 10.1016/j.bbmt.2020.01.028 32045652

[13] Jiang S Penack O Terzer T Schult D Majer-Lauterbach J Radujkovic A. Predicting sinusoidal obstruction syndrome after allogeneic stem cell transplantation with the EASIX biomarker panel. Haematologica. (2021) 106:446–53. 10.3324/haematol.2019.238790 31974195 PMC7849560

[14] Song GY Jung SH Kim K Kim SJ Yoon SE Lee HS. Endothelial activation and stress index (EASIX) is a reliable predictor for overall survival in patients with multiple myeloma. BMC Cancer. (2020) 20:803. 10.1186/s12885-020-07317-y 32831058 PMC7446202

[15] Merz A Germing U Kobbe G Kaivers J Jauch A Radujkovic A. EASIX for prediction of survival in lower-risk myelodysplastic syndromes. Blood Cancer J. (2019) 9:85. 10.1038/s41408-019-0247-z 31712595 PMC6848148

[16] Jimenez E Fontan-Vela M Valencia J Fernandez-Jimenez I Alvaro-Alonso EA Izquierdo-Garcia E. Characteristics, complications and outcomes among 1549 patients hospitalised with COVID-19 in a secondary hospital in Madrid, Spain: a retrospective case series study. BMJ Open. (2020) 10:e042398. 10.1136/bmjopen-2020-042398 33172949 PMC7656887

[17] Torres-Macho J Ryan P Valencia J Perez-Butragueno M Jimenez E Fontan-Vela M. The PANDEMYC score. An easily applicable and interpretable model for predicting mortality associated with COVID-19. J Clin Med. (2020) 9:3066. 10.3390/jcm9103066 32977606 PMC7598151

[18] Borobia AM Carcas AJ Arnalich F Alvarez-Sala R Monserrat-Villatoro J Quintana M. A cohort of patients with COVID-19 in a major teaching hospital in Europe. J Clin Med. (2020) 9:1733. 10.3390/jcm9061733 32512688 PMC7356883

[19] Collins GS Reitsma JB Altman DG Moons KG. Transparent reporting of a multivariable prediction model for individual prognosis or diagnosis (TRIPOD): the TRIPOD statement. Ann Intern Med. (2015) 162:55–63. 10.1161/CIRCULATIONAHA.114.014508 25560714

[20] Gupta RK Marks M Samuels THA Luintel A Rampling T Chowdhury H. Systematic evaluation and external validation of 22 prognostic models among hospitalised adults with COVID-19: an observational cohort study. Eur Respir J. (2020) 56:2003498. 10.1183/13993003.03498-2020 32978307 PMC7518075

[21] Miller JL Tada M Goto M Mohr N Lee S. Prediction models for severe manifestations and mortality due to COVID-19: a rapid systematic review. medRxiv. [Preprint]. (2021). 10.1101/2021.01.28.21250718 35064988

[22] Luft T Wendtner CM Kosely F Radujkovic A Benner A Korell F. EASIX for prediction of outcome in hospitalized SARS-CoV-2 infected patients. Front Immunol. (2021) 12:634416. 10.3389/fimmu.2021.634416 34248931 PMC8261154

[23] Altman DG Vergouwe Y Royston P Moons GK. Prognosis and prognostic research: validating a prognostic model. BMJ. (2009) 338:b605. 10.1136/bmj.b605 19477892

[24] Berenguer J Borobia AM Ryan P Rodriguez-Bano J Bellon JM Jarrin I. Development and validation of a prediction model for 30-day mortality in hospitalised patients with COVID-19: the COVID-19 SEIMC score. Thorax. (2021) 10:920–59. 10.1136/thoraxjnl-2020-216001 33632764 PMC7908055

