# Supplementary material for: Age-Adjusted Endothelial Activation and Stress Index for Coronavirus Disease 2019 at Admission Is a Reliable Predictor for 28-Day Mortality in Hospitalized Patients With Coronavirus Disease 2019
Source: Front Med (Lausanne). 2021 Sep 8;8:736028. doi: 10.3389/fmed.2021.736028 (PMC8455820; doi:10.3389/fmed.2021.736028)
Supplement: Supplementary file 1 [file Table_1.DOCX]

**Supplementary Table 1.** Cox regression analysis for the risk of death within 28 days in COVID-19 patients, including EASIX and baseline characteristics of the patients.

|  | **A) ILUH (derivation cohort)** | | | | **B) LPUH (validation cohort)** | | | | | | | |  |
| --- | --- | --- | --- | --- | --- | --- | --- | --- | --- | --- | --- | --- | --- |
| **Variables** | **HR (95%CI)** | **p-value** | **aHR (95%CI)** | **p-value** | | **HR (95%CI)** | | **p-value** | | **aHR (95%CI)** | | **p-value** | |
| Log_2_ EASIX | 1.63  (1.51 – 1.76) | **<0.001** | 1.55  (1.42 – 1.69) | **<0.001** | | 1.59  (1.49 – 1.68) | | **<0.001** | | 1.41  (1.31 – 1.51) | | **<0.001** | |
| Sex (male) | 1.50  (1.12 – 2.03) | **0.008** | 1.55  (1.13 – 2.13) | **0.007** | | 1.37  (1.12 – 1.67) | | **0.002** | | - | | n.s. | |
| Log_2_ Age (years) | 12.98  (7.21 – 23.37) | **<0.001** | 13.38  (7.06 – 25.38) | **<0.001** | | 13.38  (8.73 – 20.50) | | **<0.001** | | 8.86  (5.64 – 13.91) | | **<0.001** | |
| Chronic heart  disease | 2.01  (1.53 – 2.64) | **<0.001** | - | n.s. | | 1.68  (1.37 – 2.05) | | **<0.001** | | - | | n.s. | |
| Hypertension | 1.76  (1.30 – 2.37) | **<0.001** | - | n.s. | | 1.85  (1.51 – 2.28) | | **<0.001** | | - | | n.s. | |
| Chronic pulmonary  disease | 1.62  (1.18 – 2.23) | **0.003** | - | n.s. | | 1.12  (0.81 – 1.55) | | 0.480 | | Not included  (p>0.05) | | | |
| Asthma | 0.71  (0.39 – 1.30) | 0.270 | Not included  (p>0.05) | | 0.62  (0.35 – 1.11) | | 0.108 | | Not included  (p>0.05) | | | |  |
| Chronic kidney disease | 2.03  (1.38 – 2.99) | **<0.001** | - | n.s. | | 1.89  (1.48 – 2.41) | | **<0.001** | | - | | n.s. | |
| Liver cirrhosis | 1.15  (0.54 – 2.45) | 0.715 | Not included  (p>0.05) | | 1.28  (0.61 – 2.70) | | 0.518 | | Not included  (p>0.05) | | | |  |
| Neoplasm | 1.91  (1.27 – 2.90) | **0.002** | - | n.s. | | 1.43  (1.12 – 1.81) | | **0.004** | | - | | n.s. | |
| Hematological malignancy | 1.36  (0.74 – 2.49) | 0.327 | Not included  (p>0.05) | | 1.59  (1.19 – 2.13) | | **0.002** | | - | | n.s. | |  |
| Obesity | 0.62  (0.41 – 0.95) | **0.027** | Not included  (Missing values >10%) | | 1.01  (0.78 – 1.31) | | 0.955 | | Not included  (p>0.05) | | | |  |
| Diabetes | 1.23  (0.92 – 1.64) | 0.164 | Not included  (p>0.05) | | 1.39  (1.12 – 1.71) | | **0.002** | | - | | n.s. | |  |
| Dyslipidemia | 1.59  (1.09 – 2.33) | **0.017** | Not included  (Missing values >10%) | | 1.47  (1.21 – 1.79) | | **<0.001** | | - | | n.s. | |  |
| Smoker | 0.74  (0.37 – 1.47) | 0.395 | Not included  (p>0.05) | | 1.13  (0.81 – 1.57) | | 0.467 | | Not included  (p>0.05) | | | |  |
| Log_2_ Oxygen saturation  in room air | 0.07  (0.04 – 0.14) | **<0.001** | Not included  (Missing values >10%) | | 1.03  (0.76 – 1.38) | | 0.853 | | Not included  (Missing values >10%) | | | |  |
| Log_2_ Hematocrit (%) | 0.97  (0.95 – 0.99) | **0.006** | - | n.s. | | 0.64  (0.41 – 0.98) | | **0.042** | | - | | n.s. | |
| Log_2_ Lymphocyte count | 0.65  (0.54 – 0.78) | **<0.001** |  | n.s. | | 0.61  (0.55 – 0.67) | | **<0.001** | | - | | n.s. | |
| Log_2_ Neutrophil count | 1.20  (1.02 – 1.40) | **0.027** | - | n.s. | | 1.78  (1.60 – 1.98) | | **<0.001** | | 1.40  (1.24 – 1.57) | | **<0.001** | |
| Log_2_ Aspartate  Aminotransferase (AST) | 1.41  (1.22 – 1.63) | **<0.001** | - | n.s. | | 1.29  (1.19 – 1.41) | | **<0.001** | | - | | n.s. | |
| Log_2_ Alanine  Aminotransferase (ALT) | 0.88  (0.75 – 1.03) | 0.115 | Not included  (p>0.05) | | 0.88  (0.80 – 0.96) | | **0.005** | | Not included (collinearity with AST, r=0.73) | | | |  |
| Log_2_ Glucose | 1.42  (1.25 – 1.61) | **<0.001** | 1.40  (1.13 – 1.73) | **0.002** | | 2.01  (1.71 – 2.36) | | **0.001** | | 1.49  (1.25 – 1.78) | | **<0.001** | |
| Log_2_ Sodium | 19.5  (1.6 – 244.5) | **0.021** | - | n.s. | | 13.94  (2.21 – 88.07) | | **0.005** | | - | | n.s. | |
| Log_2_ Potassium | 1.73  (1.15 – 2.62) | **0.009** | - | n.s. | | 1.89  (1.15 – 3.09) | | **0.011** | | **-** | | n.s. | |
| Log_2_ C-Reactive Protein | 1.27  (1.16 – 1.40) | **<0.001** | 1.26  (1.13 – 1.41) | **<0.001** | | 1.39  (1.31 – 1.48) | | **<0.001** | | 1.21  (1.14 – 1.29) | | **<0.001** | |

**Statistics:** Values are expressed as absolute count and percentage for categorical variables, and hazard ratio and 95% confidence interval for continuous variables. Continuous variables are expressed in base-2 logarithms. First, we performed univariate Cox regression analyses. Then we performed multivariate Cox regression analyses with variables that had p-value ≤0.05, missing values ≤10%, and low collinearity between them (r <0.5), which were further selected by a stepwise forward selection method (pin <0.05 and pout <0.10). Significant differences are shown in bold.

**Abbreviations**: EASIX: endothelial activation and stress index; HR: hazard ratio; aHR: adjusted hazard ratio; 95%CI: 95% confidence interval; p-value: level of significance; n.s., not significant; ILUH: Infanta Leonor University Hospital; LPUH: La Paz University Hospital.

**Supplementary Table 2.** Cox regression analysis for the risk of death within 28 days in COVID-19 patients, including aEASIX-COVID and baseline characteristics of the patients.

|  | **A) ILUH (derivation cohort)** | | | | **B) LPUH (validation cohort)** | | | | |
| --- | --- | --- | --- | --- | --- | --- | --- | --- | --- |
| **Variables** | **HR (95%CI)** | **p-value** | **aHR (95%CI)** | **p-value** | | **HR (95%CI)** | **p-value** | **aHR (95%CI)** | **p-value** |
| Log_2_ aEASIX-COVID | 1.68  (1.57 – 1.81) | **<0.001** | 1.61  (1.49 – 1.75) | **<0.001** | | 1.64  (1.55 – 1.74) | **<0.001** | 1.51  (1.41 – 1.61) | **<0.001** |
| Sex (male) | 1.50  (1.12 – 2.03) | **0.008** | 1.46  (1.05 – 2.03) | **0.026** | | 1.37  (1.12 – 1.67) | **0.002** | 1.28  (1.03 – 1.59) | **0.025** |
| Chronic heart disease | 2.01  (1.53 – 2.64) | **<0.001** | - | n.s. | | 1.68  (1.37 – 2.05) | **<0.001** | 1.32  (1.04 – 1.63) | **0.008** |
| Hypertension | 1.76  (1.30 – 2.37) | **<0.001** | - | n.s. | | 1.85  (1.51 – 2.28) | **<0.001** | - | n.s. |
| Chronic pulmonary  disease | 1.62  (1.18 – 2.23) | **0.003** | 1.54  (1.10 – 2.15) | 0.012 | | 1.12  (0.81 – 1.55) | 0.480 | Not included  (p>0.05) | |
| Asthma | 0.71  (0.39 – 1.30) | 0.270 | Not included  (p>0.05) | | | 0.62  (0.35 – 1.11) | 0.108 | Not included  (p>0.05) | |
| Chronic kidney disease | 2.03  (1.38 – 2.99) | **<0.001** | - | n.s. | | 1.89  (1.48 – 2.41) | **<0.001** | - | n.s. |
| Liver cirrhosis | 1.15  (0.54 – 2.45) | 0.715 | Not included  (p>0.05) | | | 1.28  (0.61 – 2.70) | 0.518 | Not included  (p>0.05) | |
| Neoplasm | 1.91  (1.27 – 2.90) | **0.002** | 1.77  (1.14 – 2.75) | **0.011** | | 1.43  (1.12 – 1.81) | **0.004** | - | n.s. |
| Hematological malignancy | 1.36  (0.74 – 2.49) | 0.327 | Not included  (p>0.05) | | | 1.59  (1.19 – 2.13) | **0.002** | 1.39  (1.02 – 1.87) | **0.035** |
| Obesity | 0.62  (0.41 – 0.95) | **0.027** | Not included  (Missing values >10%) | | | 1.01  (0.78 – 1.31) | 0.955 | Not included  (p>0.05) | |
| Diabetes | 1.23  (0.92 – 1.64) | 0.164 | Not included  (p>0.05) | | | 1.39  (1.12 – 1.71) | **0.002** | - | n.s. |
| Dyslipidemia | 1.59  (1.09 – 2.33) | **0.017** | Not included  (Missing values >10%) | | | 1.47  (1.21 – 1.79) | **<0.001** | - | n.s. |
| Smoker | 0.74  (0.37 – 1.47) | 0.395 | Not included  (p>0.05) | | | 1.13  (0.81 – 1.57) | 0.467 | Not included  (p>0.05) | |
| Log_2_ Oxygen saturation  in room air | 0.07  (0.04 – 0.14) | **<0.001** | Not included  (Missing values >10%) | | | 1.03  (0.76 – 1.38) | 0.853 | Not included  (Missing values >10%) | |
| Log_2_ Hematocrit (%) | 0.97  (0.95 – 0.99) | **0.006** | - | n.s. | | 0.64  (0.41 – 0.98) | **0.042** | - | n.s. |
| Log_2_ Lymphocyte count | 0.65  (0.54 – 0.78) | **<0.001** | - | n.s. | | 0.61  (0.55 – 0.67) | **<0.001** | - | n.s. |
| Log_2_ Neutrophil count | 1.20  (1.02 – 1.40) | **0.027** | - | n.s. | | 1.78  (1.60 – 1.98) | **<0.001** | 1.30  (1.16 – 1.45) | **<0.001** |
| Log_2_ Aspartate  Aminotransferase (AST) | 1.41  (1.22 – 1.63) | **<0.001** | - | n.s. | | 1.29  (1.19 – 1.41) | **<0.001** | - | n.s. |
| Log_2_ Alanine  Aminotransferase (ALT) | 0.88  (0.75 – 1.03) | 0.115 | Not included  (p>0.05) | | | 0.88  (0.80 – 0.96) | **0.005** | Not included (collinearity with AST, r=0.73) | |
| Log_2_ Glucose | 1.42  (1.25 – 1.61) | **<0.001** | 1.42  (1.18 – 1.71) | **0.001** | | 2.01  (1.71 – 2.36) | **0.001** | 1.54  (1.28 – 1.84) | **<0.001** |
| Log_2_ Sodium | 19.5  (1.6 – 244.5) | **0.021** | 30.55  (2.20 – 424.82) | 0.011 | | 13.94  (2.21 – 88.07) | **0.005** | 8.23  (1.61 – 42.06) | **0.011** |
| Log_2_ Potassium | 1.73  (1.15 – 2.62) | **0.009** | - | n.s. | | 1.89  (1.15 – 3.09) | **0.011** | - | n.s. |
| Log_2_ C-Reactive Protein | 1.27  (1.16 – 1.40) | **<0.001** | 1.25  (1.12 – 1.38) | **0.001** | | 1.39  (1.31 – 1.48) | **<0.001** | 1.27  (1.19 – 1.35) | **<0.001** |

**Statistics:** Values are expressed as absolute count and percentage for categorical variables, and hazard ratio and 95% confidence interval for continuous variables. Continuous variables are expressed in base-2 logarithms. First, we performed univariate Cox regression analyses. Then we performed multivariate Cox regression analyses with variables that had p-value ≤0.05, missing values ≤10%, and low collinearity between them (r <0.5), which were further selected by a stepwise forward selection method (pin <0.05 and pout <0.10). Significant differences are shown in bold.

**Abbreviations**: aEASIX-COVID: age-adjusted endothelial activation and stress index for COVID-19; HR: hazard ratio; aHR: adjusted hazard ratio; 95%CI: 95% confidence interval; p-value: level of significance; n.s., not significant; ILUH: Infanta Leonor University Hospital; LPUH: La Paz University Hospital.

**Supplementary Table 3.** Sensitivity, specificity, PPV, and NPV for predicting 28-day mortality in hospitalized COVID-19 patients according to log_2-_EASIX deciles.

| **Decile** | **Cutoff** | **Sensitivity (95%CI)** | **Specificity (95%CI)** | **PPV (95%CI)** | **NPV (95%CI)** |
| --- | --- | --- | --- | --- | --- |
| **ILUH (derivation cohort)** | | | | | |
| **1** | -0.87 | 98.1 (95.2 – 99.5) | 11.7 (9.8 – 13.9) | 19.3 (16.9 – 21.7) | 96.7 (91.7 – 99.1) |
| **2** | -0.40 | 93.4 (89.2 – 96.3) | 22.9 (20.3 – 25.6) | 20.6 (18.1 – 23.3) | 94.2 (90.4 – 96.8) |
| **3** | -0.08 | 91.0 (86.4 – 94.5) | 34.5 (31.5 – 37.6) | 23.0 (20.2 – 26.0) | 94.7 (91.9 – 96.8) |
| **4** | 0.20 | 87.7 (82.5 – 91.8) | 46.0 (42.8 – 49.1) | 25.8 (22.7 – 29.2) | 94.6 (92.2 – 96.4) |
| **5** | 0.47 | 81.1 (75.2 – 86.2) | 56.7 (53.5 – 59.8) | 28.7 (25.1 – 32.5) | 93.3 (91.0 – 95.2) |
| **6** | 0.74 | 73.1 (66.6 – 79.0) | 67.1 (64.1 – 70.0) | 32.3 (28.1 – 36.7) | 92.1 (89.9 – 93.9) |
| **7** | 1.03 | 65.1 (58.3 – 71.5) | 77.5 (74.8 – 80.1) | 38.3 (33.3 – 43.6) | 91.2 (89.1 – 93.0) |
| **8** | 1.40 | 54.7 (47.8 – 61.5) | 87.4 (85.2 – 89.5) | 48.3 (41.9 – 54.9) | 90.0 (87.9 – 91.8) |
| **9** | 2.00 | 34.4 (28.1 – 41.2) | 95.2 (93.7 – 96.5) | 60.8 (51.5 – 69.6) | 87.1 (85.0 – 89.1) |
| **LPUH (validation cohort)** | | | | | |
| **1** | -0.87 | 94.7 (92.0 – 96.6) | 21.0 (18.9 – 23.2) | 25.8 (23.6 – 28.1) | 93.1 (89.8 – 95.6) |
| **2** | -0.40 | 88.3 (84.9 – 91.3) | 40.0 (37.4 – 42.6) | 30.0 (27.4 – 32.6) | 92.2 (89.8 – 94.2) |
| **3** | -0.08 | 83.5 (79.6 – 86.9) | 53.1 (50.5 – 55.7) | 34.1 (31.2 – 37.1) | 91.7 (89.6 – 93.5) |
| **4** | 0.20 | 74.8 (70.3 – 78.9) | 64.3 (61.8 – 66.8) | 37.8 (34.5 – 41.3) | 89.8 (87.7 – 91.6) |
| **5** | 0.47 | 68.4 (63.7 – 72.9) | 75.1 (72.8 – 77.3) | 44.4 (40.5 – 48.4) | 89.1 (87.2 – 90.8) |
| **6** | 0.74 | 59.2 (54.3 – 64.0) | 82.1 (80.0 – 84.1) | 49.0 (44.5 – 53.5) | 87.4 (85.5 – 89.1) |
| **7** | 1.03 | 47.6 (42.7 – 52.5) | 88.2 (86.4 – 89.8) | 53.8 (48.6 – 59.1) | 85.3 (83.3 – 87.0) |
| **8** | 1.40 | 37.4 (32.7 – 42.2) | 93.0 (91.6 – 94.3) | 60.9 (54.6 – 66.9) | 83.6 (54.6 – 66.9) |
| **9** | 2.00 | 22.8 (18.8 – 27.2) | 97.0 (96.0 – 97.9) | 69.1 (60.6 – 76.8) | 81.2 (79.3 – 83.1) |

**Abbreviations**: EASIX: Endothelial Activation and Stress Index; PPV: positive predictive value; NPV: negative predictive value; 95%CI: 95% confidence interval; ILUH: Infanta Leonor University Hospital; LPUH: La Paz University Hospital.

**Supplementary Table 4.** Sensitivity, specificity, PPV, and NPV for predicting 28-day mortality in hospitalized COVID-19 patients according to log2-aEASIX-COVID deciles.

| **Decile** | **Cutoff** | **Sensitivity (95%CI)** | **Specificity (95%CI)** | **PPV (95%CI)** | **NPV (95%CI)** |
| --- | --- | --- | --- | --- | --- |
| **ILUH (derivation cohort)** | | | | | |
| **1** | 4.89 | 99.5 (97.4 – 100.0) | 12.0 (10.1 – 14.2) | 19.5 (17.2 – 22.0) | 99.2 (95.4 – 100.0) |
| **2** | 5.47 | 97.6 (94.6 – 99.2) | 23.8 (21.2 – 26.6) | 21.6 (19.0 – 24.3) | 97.9 (95.2 – 99.3) |
| **3** | 5.87 | 94.3 (90.3 – 97.0) | 35.2 (32.2 – 38.3) | 23.8 (21.0 – 26.8) | 96.7 (94.2 – 98.3) |
| **4** | 6.18 | 91.5 (86.9 – 94.9) | 46.8 (43.6 – 49.9) | 26.9 (23.7 – 30.3) | 96.3 (94.1 – 97.8) |
| **5** | 6.47 | 87.3 (82.0 – 91.4) | 58.0 (54.8 – 61.1) | 30.8 (27.2 – 34.7) | 95.5 (93.5 – 97.0) |
| **6** | 6.81 | 80.2 (74.2 – 85.3) | 68.6 (65.6 – 71.5) | 35.4 (31.1 – 39.9) | 94.2 (92.2 – 95.8) |
| **7** | 7.09 | 71.2 (64.6 – 77.2) | 78.8 (76.2 – 81.4) | 41.9 (36.8 – 47.2) | 92.7 (90.8 – 94.4) |
| **8** | 7.51 | 57.1 (50.1 – 63.8) | 88.0 (85.8 – 89.9) | 50.4 (43.9 – 56.9) | 90.5 (88.5 – 92.3) |
| **9** | 8.24 | 37.7 (31.2 – 44.6) | 96.0 (94.5 – 97.1) | 66.7 (57.5 – 75.0) | 87.8 (85.7 – 89.7) |
| **LPUH (validation cohort)** | | | | | |
| **1** | 4.89 | 97.8 (95.9 – 99.0) | 19.3 (17.3 – 21.5) | 26.1 (23.9 – 28.3) | 96.8 (94.0 – 98.5) |
| **2** | 5.47 | 94.7 (92.0 – 96.6) | 36.0 (33.5 – 38.6) | 30.1 (27.6 – 32.6) | 95.9 (93.8 – 97.4) |
| **3** | 5.87 | 89.6 (86.2 – 92.3) | 52.3 (49.6 – 54.9) | 35.3 (32.4 – 38.3) | 94.5 (92.7 – 96.0) |
| **4** | 6.18 | 84.0 (80.1 – 87.4) | 64.2 (61.6 – 66.7) | 40.5 (37.2 – 43.9) | 93.2 (91.5 – 94.7) |
| **5** | 6.47 | 76.2 (71.8 – 80.2) | 73.1 (70.7 – 75.4) | 45.1 (41.4 – 48.9) | 91.4 (89.6 – 92.9) |
| **6** | 6.81 | 65.3 (60.5 – 69.9) | 81.6 (79.5 – 83.6) | 50.8 (46.4 – 55.1) | 89.0 (87.2 – 90.6) |
| **7** | 7.09 | 56.6 (51.6 – 61.4) | 87.2 (85.4 – 88.9) | 56.3 (51.4 – 61.1) | 87.4 (85.5 – 89.0) |
| **8** | 7.51 | 42.0 (37.2 – 46.9) | 92.7 (91.3 – 94.0) | 62.7 (56.7 – 68.4) | 84.6 (82.7 – 86.4) |
| **9** | 8.24 | 23.5 (19.5 – 27.9) | 97.3 (96.3 – 98.1) | 71.9 (63.5 – 79.2) | 81.4 (79.5 – 83.2) |

**Abbreviations**: EASIX: Endothelial Activation and Stress Index; aEASIX-COVID: age-adjusted EASIX for COVID-19; PPV: positive predictive value; NPV: negative predictive value; 95%CI: 95% confidence interval; ILUH: Infanta Leonor University Hospital; LPUH: La Paz University Hospital.
